# Interventional Techniques for the Management of Cancer-Related Pain: Clinical and Critical Aspects

**DOI:** 10.3390/cancers11040443

**Published:** 2019-03-29

**Authors:** Geana Paula Kurita, Per Sjøgren, Pål Klepstad, Sebastiano Mercadante

**Affiliations:** 1 Palliative Research Group, Department of Oncology, Rigshospitalet Copenhagen University Hospital, 2100 Copenhagen, Denmark; per.sjoegren@regionh.dk; 2 Multidisciplinary Pain Centre, Department of Neuroanaesthesiology, Rigshospitalet Copenhagen University Hospital, 2100 Copenhagen, Denmark; 3 Department of Clinical Medicine, Faculty of Health and Medical Sciences, University of Copenhagen, 2200 Copenhagen, Denmark; 4 Department Intensive Care Medicine, St. Olavs University Hospital, 7006 Trondheim, Norway; pal.klepstad@ntnu.no; 5 Department of Circulation and Medical Imaging, Norwegian University of Science and Technology, 7491 Trondheim, Norway; 6 Main Regional Center for Cancer Pain and Supportive/Palliative Care, Anesthesia and Intensive Care Unit, and Pain Relief and Palliative Care Unit, La Maddalena Cancer Center, 90145 Palermo, Italy; terapiadeldolore@lamaddalenanet.it

**Keywords:** cancer-related pain, neuraxial analgesia, vertebroplasty, sympathetic blocks, peripheral nerve blocks, cordotomy

## Abstract

Interventional techniques to manage cancer-related pain may be efficient treatment modalities in patients unresponsive or unable to tolerate systemic opioids. However, indication and selection of the right technique demand knowledge, which is still incipient among clinicians. The present article summarizes the current evidence regarding the five most essential groups of interventional techniques to treat cancer-related pain: Neuraxial analgesia, minimally invasive procedures for vertebral pain, sympathetic blocks for abdominal cancer pain, peripheral nerve blocks, and percutaneous cordotomy. Furthermore, indication, mechanism, drug agents, contraindications, and complications of the main techniques of each group are discussed.

## 1. Introduction

Nociception is the process by which information about actual or potential tissue damage is conveyed through the spinothalamic tract to the brain, where the information is processed and interpreted, and the descending pain modulatory system is activated [1]. The interruption of nociceptive pathways at peripheral and central levels can be an alternative treatment approach for patients with cancer-related pain. It can be done by interventional nondestructive or neuroablatory techniques and is particularly indicated for the management of cancer-related pain with an unsatisfactory response to strong systemic opioids and adjuvant analgesics and/or in patients with intolerable side effects of systemic analgesics [2]. Such pain is often referred to as refractory pain.

Most patients with cancer-related pain obtain satisfactory pain relief from medications that are referenced in the WHO analgesic ladder [3]. However, it has been estimated that approximately 2–5% of patients with advanced cancer have inadequate control of pain with systemic analgesics [4]. These patients may alternatively benefit from interventional techniques such as neuraxial analgesia, minimally invasive procedures for vertebral pain, sympathetic blocks for abdominal cancer pain, peripheral nerve blocks, and percutaneous cordotomy. The successful use of these techniques depends on the selection of the right therapy for the right patient.

A careful assessment of the patient is important to achieve satisfactory outcomes. This assessment includes patient characteristics, previous treatments, pain characteristics and mechanisms, patients’ preference, and skills and logistics to perform the selected invasive technique. Moreover, interventional techniques imply risks for complications. Therefore, potential benefits and harms should be considered for each patient [2].

The present article aims at summarizing current evidence and improving specialist clinicians’ knowledge regarding the five most essential classes of interventional techniques to treat cancer-related pain: Neuraxial analgesia, minimally invasive procedures for vertebral pain, sympathetic blocks for abdominal cancer pain, peripheral nerve blocks, and percutaneous cordotomy. For each technique, we will briefly review indications, mechanisms, drug agents, current evidence, contraindications, and complications.

## 2. Interventional Techniques

### 2.1. Neuraxial Analgesia for Cancer Pain

When systemic analgesics are exhausted, the selection of patients for neuraxial therapy (epidural and intrathecal routes) is often based on location and mechanism of pain—especially when considering the benefits of adding local anesthetics [5,6] or other adjuvants as clonidine/ketamine to opioids [7,8]. Patients with cancer-related pain and longer survival expectancy (>3 months) may benefit from neuraxial therapies using implantable systems as a permanent intrathecal catheter and subcutaneous pump, while patients with shorter life expectancy may be treated with epidural therapy with implanted system as a catheter or port-a-catch connected to an external PCA pump [9]. The selection of device type usually depends on survival expectancy, patients’ needs, and costs. The mechanism of neuraxial analgesia is based on opioid binding to its receptor in the spinal cord, which reduces or blocks the nociceptive signal conduction. Opioids may also interfere with descending pathways and modulate the pain pathway in the midbrain. However, other anesthetic agents as bupivacaine, which has a long duration of action and low toxicity and costs, may be used as the main spinal therapy or in addition to opioids. Some examples of other agents are clonidine, ketamine, and neostigmine, which may improve analgesia and reduce opioid doses [9].

A systematic review [10] analyzed the evidence regarding analgesic efficacy and side effects of neuraxial drug administration in adult patients with cancer-related pain and found nine RCTs, which were divided in four groups: (A) Sufentanil or morphine combined with adjuvant analgesic (bupivacaine, clonidine, ketamine, neostigmine, or midazolam) compared with an opioid alone (n = 4); (B) morphine or aqueous phenol in bolus compared with continuous administration (n = 2); (C) ziconotide compared with placebo (n = 1); and (D) morphine or hydromorphone with or without adjuvant analgesics compared with comprehensive medical management (n = 2). The studies had different designs and methodologies, which excluded a meta-analysis. Limitations of the studies analyzed included lack of power sample calculation, flaws in randomization, problematic control groups, large dropouts, small study samples, patient crossing over between the treatment arms, meagre pain mechanism descriptors, and in some studies, exaggerated focus on the company-sponsored implantable systems—all of which reduced external, as well as internal, validity. Due to these issues, the quality of evidence was judged as very low. However, all studies demonstrated better pain control for all interventions analyzed. Side effects were described, and there seemed to be few significant differences in favor of the tested interventions. We conducted a search update for the neuraxial administration of analgesics (Nov 2018) following the same methodology as the systematic review mentioned [10] and found two additional articles. The first is a double-blinded cross-over study (n = 24), which was performed to investigate the effect of intrathecal dexmedetomidine as an add-on therapy to intrathecal analgesia in patients with refractory cancer-related pain. Pain intensity and morphine consumption were lower with the addition of dexmedetomidine [11]. The second study is a double-blind RCT (n = 36), which compared the efficacy and safety of an intrathecal continuous infusion of morphine and ropivacaine versus intrathecal morphine alone for cancer-related pain. Pain intensity and quality of life improved with the addition of ropivacaine [12]. However, major methodological flaws were attached to both studies including lack of statistical power. Thus, the addition of two recent RCTs did not change the conclusion of the previous published review [10].

Contraindications to neuraxial therapy may exist in fragile patients, and complications of varying severity are described in the literature [4]. They include harm to spinal cord or fibers during punction and catheter placement, headache after dural puncture, epidural hematoma, infections, meningitis, and catheter migration, among others [13]. Therefore, a limited number of patients with cancer-related pain are candidates for neuraxial therapy, which also reflects the difficulties in performing high quality RCTs.

### 2.2. Minimally Invasive Procedures for Vertebral Pain: Vertebroplasty, Kyphoplasty, Radiofrequency Ablation, and Cryoablation

Percutaneous vertebroplasty (PV), kyphoplasty (KP), radiofrequency ablation (RFA), and cryoablation (CA) are minimally invasive procedures indicated for the relief of vertebral bone pain in patients with metastatic lesions and/or compression fracture without neurologic sequelae. Despite the reported effectiveness and low risk associated with these interventions, availability of trained staff, lack of precise indications, and the high costs render the access to minimally invasive procedures sparse [14]. Thus, the use of these procedures should be reserved for a selected cohort of patients with severe and disabling cancer-related back pain refractory to systemic analgesic treatment.

PV usually involves percutaneous injection of a cement, polymethylmethacrylate into the vertebral bodies, which can provide a mechanical stabilization of the lesion/compression fracture, increase bone strength and alleviate pain. KP is a variation of PV, which is performed by inflating a balloon in the vertebral body to make an empty space where the cement can be placed to correct vertebral height and kyphotic irregularities. In RFA, bone tumor or metastases are ablated using the heat generated from medium frequency alternating current. CA is an alternative to RFA in patients with metastatic bone disease and it is performed using cryoprobes, through which cooled, thermally conductive fluids are circulated. The area of tissue destruction created by this technique can be delimited more effectively by computed tomography than RFA. This is a potential advantage when treating tumors adjacent to critical anatomical structures. These techniques have gained worldwide application for back pain due to osteoporotic and malignant vertebral collapses refractory to conservative treatment [15]. However, the role of these techniques remains controversial, particularly in patients with advanced cancer.

Several trials suggested a possible therapeutic role of these procedures, but existing data are often limited to series with a low number of patients, retrospective designs, or mixed populations. The low number of patients and the poor quality of data have not yet provided evidence for efficacy in cancer patients with vertebral tumors or metastases. Many factors confounded the interpretation of data and included a low accrual rate, patients with different types of fractures, unclear evaluation of fractures, other causes of pain, problematic sham designs, and lack of proper clinical examinations to determine the causes of pain [16]. A systematic review performed in 2015 analyzed the evidence to support the performance of percutaneous procedures to treat vertebral pain due to cancer. Articles were selected considering interventional techniques compared with analgesic drugs or sham procedures, but noncomparative observational investigations with a minimum set of 50 patients were also analyzed due to the sparse number of controlled studies. The authors found five studies that met their inclusion criteria: Two regarding KP and three PV. Out of these, only KP had a recommendation in favor of performing this intervention in patients with vertebral tumors or metastases. However, the studies presented several weaknesses and low-quality of study designs, which reduced significantly the strength of recommendation [17]. In an update of that review (Nov 2018) following the same methodology [17], we identified another systematic review performed in 2014 with different characteristics and inclusion criteria, which also assessed treatment safety and effectiveness of vertebroplasty and kyphoplasty for treatment of cancer-related vertebral fractures and found only six RCTs among 150 selected studies. They compared vertebral augmentation with traditional management and treatments for tumor control. Pain intensity, pain-related disability, and health-related quality of life improved following kyphoplasty compared to usual care [18]. Recent literature after 2015 still lacks substantial data [18]. The heterogeneity of study designs, outcomes, and populations still suggests that current literature provided inconsistent data and further studies should delineate confounding variables. In a recent document of the European Association for Palliative Care [17], these weaknesses and the low quality of study designs were confirmed. Recommendations for performing RFA are weak. Further RCTs of PV and KP in patients with cancer-related pain are required to improve the strength of evidence available to recommend these procedures on large scale.

Contraindications for these invasive procedures are coagulopathy, neurological symptoms by vertebral compression or tumor encroachment on or within the spinal cord, complete collapse of the vertebra, presence of systemic or local infections, and certain types of lesions, such as osteoblastic metastasis [18,19]. Complications are of concern [20,21], as cement leakage occurred in 20–70% of patients [22,23,24,25], and cement embolism was found in 26% of patients [26]. Moreover, symptomatic vertebral fracture may be a common adverse event after KP, but there is a higher risk of vertebral fracture after VP than KP [27]. KP seems to be at a lower risk of cement extravasation [15]. Thus, these procedures carry risks of substantial harm [26]. Finally, the relationship of PV, radiotherapy, and surgery remains unresolved [15]. For the other techniques, no such data are available.

### 2.3. Sympathetic Blocks for Abdominal Cancer-Related Pain: Celiac Plexus Block and Superior Hypogastric Plexus Block

The neurolytic blockade of sympathetic pathways at different levels are indicated to patients with abdominal pain with a visceral mechanism, although abdominal pain often has concomitance of different pathophysiological mechanisms [28]. The celiac plexus originates from the sympathetic fibers of the splanchnic nerves raising from T5 to T12. It is the main target point where fibers to the upper abdominal area can be blocked by one injection. Splanchnic blocks may be an alternative to celiac block and may produce responses in those failing to respond to celiac blocks when the target area is invaded by the tumor [29].

Celiac plexus block (CPB) was claimed for the treatment of cancer-related pain originating from upper abdominal viscera, while the superior hypogastric plexus block (SHPB) is targeted for lower abdominal pain. These blocks interfere with neural conduction to abolish or reduce pain. Afferent and efferent conduction may be disrupted by injection of local anesthetics [30]. Different techniques were developed in an attempt to achieve the best analgesia with less complications [31].

A systematic review performed in 2014 investigated the evidence to support the treatment of adult patients with abdominal cancer-related pain with sympathetic blocks. The analysis included papers on sympathetic blocks compared with analgesic drugs/placebo and was conducted in adult patients with cancer-related pain. Twenty-seven studies were analyzed; 15 were RCTs, but only four were double-blind [32]. It was concluded that there was a strong evidence that CPB, performed by different techniques, provides good analgesic effects and/or reduces opioid consumption and some opioid-induced adverse effects in comparison with conventional analgesic treatment [33,34,35,36,37,38,39,40,41,42,43,44]. However, two studies performed with different techniques [38,40] found that quality of life and survival did not seem to be affected. An update of the aforementioned systematic review (Nov 2018) following the same methodology [32] did not find new evidence.

The most appropriate timing for performing CPB is still controversial. It was suggested that CPB should be performed early, even in the presence of mild pain or in patients receiving low doses of opioids [34,36,37,38]. However, this statement remains unproven [41]. Some authors reported that controlling pain with analgesics and then performing the CPB was more effective than an early block followed by pharmacotherapy [45]. In patients with abdominal-pelvic pain, who were potential candidates to CPB or SHPB, the analgesic treatment with low doses of opioids was effective in all patients [45]. It is likely that the efficacy of CPB depends on individual anatomical distribution, which is likely to be distorted with the local progression of the tumor. When tumor spread involves other somatic areas such as peritoneum or diaphragm, it is likely that the block will be less successful, as CPB can block the sympathetic pathways for visceral pain only. Pain may worsen due to the involvement of areas outside the territory dependent on sympathetic pathways. Indeed, no early procedure may prevent the subsequent neural and somatic structure involvement, which seems to be unpredictable [46]. As pain and disease progression are unforeseeable, an early block could be disproportionate in many circumstances. On the other hand, a late block, when somatic structures are involved, or celiac area is distorted, is likely to be unsuccessful. Thus, the decision to perform a CPB should be based on detailed information regarding patient condition, aim of the treatment (reduction of opioid consumption or opioid adverse effects), and patient participation in decision making.

Data regarding SHPB is sparse, as only one controlled clinical study has been produced [47]. The origin of pelvic cancer-related pain, for which the technique is claimed, is even more complex, because different pain mechanisms may coexist due to the various overlapping structures including muscles and nerves [45]. It is likely that this block of sympathetic pathways is unable to abolish the nociception. According to data from literature, there is a weak recommendation in favor of using this intervention.

General contraindications for neurolytic blocks of sympathetic pathways include tumor invasion into the insertion site, coagulopathy, systemic or localized infection, complicated anatomy, and bowel obstruction [2,48]. Reported complications are back pain, orthostatic hypotension, diarrhea, retroperitoneal hematoma, bladder or ureteral injury, and inadvertent somatic nerve damage [2,48]. Different from neurolytic somatic blocks, CPB is claimed to be safe because neurological complications are unlikely and mostly transient when performed with CT guidance.

### 2.4. Peripheral Nerve Blocks: Paravertebral Blocks, Blocks in the Head Region, Plexus Blocks, and Intercostal Blocks

A logical approach for pain otherwise difficult to treat is to block peripheral nerves with local anesthetics and thereby block the signaling of nociceptive input to the central nervous system. The selection of which nerve block to apply is dependent on the source of pain. Therefore, the physicians involved in administering peripheral nerve blocks must have good knowledge of the innervation supplied from each nerve and the anatomical localization and access to the nerve. Peripheral blocks currently reported that are applied to cancer-related pain include paravertebral blocks, brachial plexus blocks, blocks of nerves in the head region, and intercostal nerves.

The drugs used for nerve blocks are local anesthetics, which have to be applied continuously or intermittently because of a limited duration of action. Local anesthetic in use are lidocaine, bupivacaine, and ropivacaine, among others. The analgesic effect is similar, while toxicity may differ in relation to cardiac events and other local anesthetic toxic events. However, if the local anesthetic is given within the recommended dosages, the risk for toxicity is minimal. The duration of each local anesthetic action differs. Lidocaine is typically considered a local anesthetic of intermediate action (1.5–3 h), while bupivacaine and ropivacaine are long-action local anesthetics (4–18 h) [49]. The duration may be altered by additives, such as epinephrine or clonidine, and varies between different localization of the nerve block [50]. Therefore, for long-term therapy with local anesthetics, bupivacaine or ropivacaine is best suited, of which bupivacaine by tradition is the most frequently reported agent in use. Some reports on the use of peripheral nerve block applied neurolytic agents such as glycerol, phenol, or alcohol. These agents irreversibly destroy the nerve and only need a single injection for a long-term block of the signaling of the nerve impulse.

The evidence for use of peripheral blocks is limited. A systematic review published in 2015 identified 16 papers, which reported a total of 79 cases [51]. The nerve blocks were paravertebral blocks (10 cases), block in the head region (two cases), plexus blocks (13 cases), intercostal block (43 cases), and other (11 cases). Most cases experienced pain relief, many for several weeks. Except for catheter displacement and one report of toxic effects associated with injections of butamben, an ester local anesthetic not used in any other reports, and there were no adverse effects. This systematic review identified no controlled trials. An update (Nov 2018) using the same methods as in the systematic review mentioned [50] identified additionally two cases receiving interscalene continuous plexus blocks [52], one case with plexus phenol neurolysis [53], two cases with alcohol paravertebral neurolysis [54], and one case with a femoral nerve block [55].

In summary, the evidence for use of peripheral blocks for cancer-related pain is still anecdotal. Still, the published cases illustrate the potential benefit from use of peripheral blocks. New techniques such as ultrasound identification of nerves, now routinely applied for use of local anesthesia during surgical procedures, will ease the placement of catheters for administration of local anesthetics. Moreover, techniques to ensure that catheters do not dislodge can be improved. Thus, physicians involved in treatment of cancer-related pain could benefit from collaboration with anesthesiologist involved in orthopedic surgery [56]. At the time technical issues associated with continuous administration of local anesthetics improve, new studies should compare the use of peripheral blocks to current standard treatment of cancer-related pain. Such studies can compare patients’ preferences in relation to inconveniences related to the use of local anesthetics (potential motor or sensory deficits and to carry an infusion pump) against opioid adverse effects.

Local anesthetics have adverse effects related to the potential drug toxicity, to the effects from the nerve block in itself or as a complication to the procedure. Toxicity is not a clinical issue given that the local anesthetic is administered at recommended doses and not inadvertently injected intravascularly. However, because of a low, but still present risk of acute toxicity (cardiac toxicity and seizures) due to an intravascular injection, physicians administering nerve blocks should have the knowledge and equipment available to handle such incidents. Complications related to the effect from local anesthetic is not a major clinical issue. If, for instance, a plexus nerve block is given in a dose that paralyses the arm, the local anesthetic dose can simply be reduced, or the infusion discontinued, and motor function will resolve. Also, complications related to the procedure of insertion is not reported for patients with cancer-related pain. There may be a risk for nerve injury resulting in neuropathic pain but given that the procedure is performed by skilled personnel using adequate techniques, this risk is small. Moreover, peripheral block is not associated with risk such as meningitis, epidural abscesses or hematomas feared by central blocks. Neurolytic peripheral blocks, on the other hand, will result in permanent disability if wrongly performed, and may—if correctly performed—after some time, result in neuropathic pain caused by reinnervation. Therefore, neurolytic blocks should not be the first-choice treatment. It should be used rarely and only in patients with expected short survival time (<3 months).

### 2.5. Percutaneous Cordotomy

Percutaneous cordotomy by radiofrequency has become a viable choice due to advances on fluoroscopic equipment and the possibility of performing the procedure under sedation instead of general anesthesia (usual procedure in open cordotomy), which represents an advantage to fragile patients with advanced cancer. However, this intervention causes an irreversible disruption of the nervous conduction and, therefore, risks and consequences should be examined carefully. The selection of patients with cancer for this procedure is based on presence of malignant unilateral pain refractory to systemic treatment and other nondestructive interventions, life expectancy of few years or less considering the possibility of pain recurrence (lesioned nerves may regrow, and neuropathic pain may return), and anatomic location of the intervention that permits a safe procedure [2,57]. It is recommended to treat unilateral pain caused by malignant pleural mesothelioma, breast cancer, brachial plexus pain related to pancoast tumor pressure/infiltration, or unilateral leg pain. This treatment can also be used in bilateral cancer-related pain, but bilateral interventions increase the risk of complications and mortality [58].

Percutaneous cordotomy is a thermal lesion caused by radiofrequency electric current in spinothalamic tract contralateral to the side of the pain, interrupting conduction fibers and selectively eliminating pain and temperature sensation from the affected half of the body. Its mechanism is unclear, but alteration on descending impulse or dorsal horn modulation, damage of C fibers and immunomodulatory effect may all play a role on pain transmission [2]. In conventional radiofrequency, the needle tip is heated between 80 °C and 90 °C, while in pulsed radiofrequency, the temperature is kept below 43 °C to avoid tissue harm [2]. Radiofrequency lesions may be done at several sites, but main entry point is located over the C1/2 intervertebral foramen on the opposite side of the source of pain and may only be suitable to treat pain below the level of the C4 dermatome [58].

Retrospective and uncontrolled studies suggested that percutaneous cordotomy is effective in reducing pain, and decreasing or discontinuing opioid use [59,60,61]. However, we performed a search using the words cordotomy, pain, and cancer on PubMed (Nov 2018). The search revealed a lack of controlled studies and very few recent prospective studies about the effects of this treatment in cancer-related pain as demonstrated by the limited number of case studies/series [62,63,64] and narrative [65] and systematic reviews [66] published in the last six years. The case-series showed positive effects of percutaneous cordotomy alone or in combination with intrathecal neuromodulation in 45 retrospective and five prospective cases involving cancer-related pain [63,64]; the case study of two patients reported positive effects of percutaneous cordotomy in only one [62], while the systematic review of case-series involving 160 patients pointed out that the procedure seemed to be effective and safe. However, evidence was very limited in terms of quantity and quality [65,66].

The side effects of this procedure include numbness and dysesthesias below the level of the lesion. Contraindications are abnormal coagulation, infection, severe respiratory dysfunction, and advanced disease that hinders positioning the patient correctly during the procedure [58]. Bilateral cordotomy procedures are generally not recommended at the high cervical region due to risk of respiratory depression. Possible complications include hemiparesis, respiratory irregularities, bladder and bowel dysfunction (usually temporary), and ataxia [57].

## 3. Considerations

The present review is not exhaustive regarding interventional techniques to the management of refractory cancer-related pain. However, other invasive techniques, e.g., midline myelotomy and stereotactic cingulotomy, are otherwise promising based on very few up-to-date small size series [67,68]. Despite the limited scientific evidence, all interventional techniques described in the present review seem to provide pain relief to patients with cancer-related pain of difficult management or of intractable nature (Table 1). However, careful attention of technique and equipment selection, dexterity to perform the procedure, sufficient logistics and staff skills, appropriate indication based on accurate clinical diagnosis and well-defined/localized pain, assessment of benefits/risks and contemplation of patient’s wish/agreement are indispensable. In addition, indication for cancer-related pain should also consider patient’s health status, estimate of survival time, and needs in order to avoid complications and qualify proper intervention selection.

## Figures and Tables

**Table 1:** Summary of the systematic reviews accessed.

Reviews	Neuraxial analgesia [10]	Minimally invasive procedures for vertebral pain [17]	Sympathetic blocks for abdominal cancer pain [32]	Peripheral nerve blocks [51]	Percutaneous cordotomy [66]
Number of studies analysed	9 (RCTs)	5 (1 RCT, 4 case-series)	15 (RCTs)	16 (case-histories/series)	9 (case-series)
Technique, number of studies (sample) size	▪ Opioid and adjuvant analgesic compared with neuraxial opioid alone, 4 (n = 22–85) ▪ Single drug in bolus compared with continuous administration, 2 (n = 28–78) ▪ Single drug compared with placebo, 1 (n = 108) ▪ Opioid with/without adjuvant analgesic compared with other medical management, 2 (n = 143–154)	▪ Radiofrequency ablation, 0 ▪ Cryoablation, 0 ▪ Kiphoplasty, 1 RCT (n = 134); 1 case-series (n = 65) ▪ Vertebroplasty, 3 case-series (n = 52, 106, 128)	▪ Celiac plexus block, 14 (n = 20–137) ▪ Superior Hypogastric plexus block, 1 (n = 50)	▪ Paravertebral blocks, 3 (n = 10) ▪ Blocks in the head region, 2 (n = 2) ▪ Plexus blocks, 6 (n = 13) ▪ Intercostal blocks, 2 (n = 43) ▪ Others, 4 (n = 11) (1 study had had different types of nerve blocks)	▪ Unilateral cervical, 9 (n = 160)
Follow-up range	▪ 10–169 days	▪ Kiphoplasty: RCT, 1 month Case-series, 21 months ▪ Vertebroplasty: 6 weeks–17 months	▪ Celiac plexus block: 1 month–death ▪ Superior Hypogastric plexus block: 3 months	▪ 5 days–death	▪ 2–28 days or more
Pain relief	▪ In all studies (comparisons of groups’ mean scores)	▪ In all studies (majority by comparisons of mean scores) ▪ One vertebroplasty series reported analgesic efficacy of 86%–92%	▪ In the majority (comparisons of groups’ mean scores)	▪ In the majority	▪ In the majority
Adverse effects	▪ Few significant differences	▪ Kiphoplasty: RCT groups were similar Case-series: 8%–12.1% ▪ Vertebroplasty: 2.3%–8.5%	▪ Celiac plexus block: decreased opioid-induced adverse effects ▪ Hypogastric plexus block: no difference	▪ Very few	▪ Several, but mostly transient
Evidence quality	Low	Low	High—celiac plexus block Low—hypogastric plexus block	Low	Low
Recommendation in favour or against the interventions	Weak in favour	Weak in favour of kyphoplasty Weak against vertebroplasty	Strong in favour of celiac plexus block Weak in favor of hypogastric plexus block	Weak in favour	Weak in favour
